# Validity and Reliability of an Arabic Version of the Survey Instrument for Natural History, Aetiology and Prevalence of Patellofemoral Pain Studies: A Cross-Sectional Study

**DOI:** 10.3390/ijerph21060732

**Published:** 2024-06-05

**Authors:** Mohammad Z. Darabseh, Aseel Aburub, Thamer A. Altaim, Badrieh Al Abbad, Khaldoon Bashaireh

**Affiliations:** 1 Department of Physiotherapy, School of Rehabilitation Sciences, The University of Jordan, Amman 11942, Jordan; 2 Department of Physiotherapy, Faculty of Allied Medical Sciences, Applied Science Private University, Amman 11931, Jordan; aburubaseel@gmail.com; 3 Department of Physiotherapy, Faculty of Allied Medical Sciences, Al-Ahliyya Amman University, Amman 19628, Jordan; thamerpt@live.co.uk; 4 School of Allied Health Professions, Keele University, Newcastle Under Lyme ST5 5BG, UK; b.al.abbad@keele.ac.uk; 5 Department of Special Surgery, Division of Orthopaedics, Faculty of Medicine, Jordan University of Science and Technology, Irbid 22110, Jordan; bashaireh@just.edu.jo

**Keywords:** patellofemoral pain, cross-cultural adaptation, reliability, validity, SNAPPS, knee pain, physiotherapy

## Abstract

Background and Objectives: Knee pain, specifically patellofemoral pain (PFP), may lead to limitations in physical activity and social participation. Identifying knee pain that is attributed to PFP is not an easy job for healthcare professionals. To overcome this issue, The Survey Instrument for Natural History, Aetiology and Prevalence of Patellofemoral Pain (SNAPPS), which is a self-reporting questionnaire instrument, was designed to identify PFP in many languages. However, the Arabic version of the SNAPPS is not validated yet. This study was performed to assess the validity and reliability of the Arabic version of the SNAPPS (A-SNAPPS). Materials and Methods: A cross-sectional study was conducted to achieve the study goals. To assess reliability, 38 participants were asked to complete the A-SNAPPS two times on the same day with a 30 min break in between. Convergent validity of the A-SNAPPS was assessed by exploring the correlations of the SNAPPS total score with the visual analogue scale (VAS) scores, including VAS for usual pain, VAS for worst pain, and VAS for pain during activities such as jumping, running, ascending and descending stairs, and squatting. Results: The validity test findings suggested that SNAPPS has a strong correlation with the VAS during ascending and descending stairs (r = 0.71) and moderate correlations during jumping (r = 0.54) and squatting (r = 0.57). The test–retest reliability ICC was 0.92, indicating a very strong test–retest reliability of the A-SNAPPS. Conclusions: The A-SNAPPS was cross-culturally adapted and validated, demonstrating very strong reliability.

## 1. Introduction

Lower limbs are most likely to get injured amongst young athletes and physically active individuals [1]. Injuries are mostly evident in the knee joint; this is common in people who take part in regular exercise/physical activities [1]. It is essential to understand and to get familiar with the different knee injuries and/or diseases to identify the appropriate management and prevention techniques. Patellofemoral pain (PFP) usually presents as diffuse anterior knee pain, typically with activities such as running, squatting, and stair ascent and descent [2]. It has been shown that PFP was the most commonly reported running-induced injury leading to pain with the knee the most common location of injury [3]. This pain is translated into anterior knee pain, which is a common symptom with no pathognomonic signs at the time of diagnosis [4]. There are no established clinical guidelines for diagnosing PFP; PFP is usually diagnosed by ruling out other knee conditions [4]. Previously, studies have used different clinical tests and/or medical notes to help with diagnosis, but these were considered time-consuming, of high cost, and inconvenient for participants [5]. Therefore, a self-reported questionnaire, “The Survey Instrument for Natural History, Aetiology and Prevalence of Patellofemoral Pain (SNAPPS)”, was developed as a specific PFP tool to help assess PFP in people presenting with anterior knee pain [5]. This questionnaire identifies people with PFP by discriminating between people with PFP and without PFP in the community [5]. SNAPPS reported high test and retest reliability to discriminate anterior knee pain with or without the PFP condition, in addition to high sensitivity and specificity (>90%) [5]. The questionnaire was designed based on the pain map in the knee and its clinical features. SNAPPS has been clinically implemented and studied in different locations including China, Africa, and a number of European countries, and it has been translated into different languages [5,6,7,8,9]. There is an increased demand for international research for a diverse culture and population that highlights the need to validate self-reported tools/instruments to a wider community. The SNAPPS was initially developed in the English language and has been translated and validated into different languages [5]. 

Cross-cultural adaptation of health questionnaires is a widely recognized necessity to ensure the accuracy and reliability of health assessments across different languages and cultures. Studies have shown successful adaptation of similar tools in various languages, such as Chinese, Spanish, and French, highlighting the methodological rigor required to maintain the validity of the instrument [10,11]. For instance, the translation and validation process often involves forward and backward translation, expert committee review, and pretesting in the target population to ensure cultural relevance and comprehension [12]. This comprehensive process ensures that the tool not only maintains its psychometric properties but also resonates well within the cultural context of the target population. Therefore, adapting the SNAPPS questionnaire into Arabic is a crucial step to facilitate accurate and reliable assessment of patellofemoral pain in Arabic-speaking populations, contributing to better clinical outcomes and research comparability across different cultural settings [13].

SNAPPS was recently translated to the Arabic language; however, to this date, the SNAPPS has not been validated into the Arabic language. Arabic is a well-known language that is spoken across twenty-five countries; it is considered one of the official languages of the World Health Organization (WHO), along with English, French, Spanish, Russian, and Chinese [14]. Even though most of the healthcare professionals in the Arab world have a basic understanding of written English, it will be challenging to utilize self-reported tools like the SNAPPS in an Arabic-speaking population without validating the tool in Arabic. Therefore, the aim of this study was to cross-culturally adapt the questionnaire for Arabic-speaking countries.

## 2. Methods

### 2.1. Study Design

A cross-sectional study was conducted to achieve the study goals. The translation process occurred from March 2021 to January 2022, and the test–retest reliability and validity checks were performed in December 2023 and January and February 2024.

### 2.2. The SNAPPS

The SNAPPS was developed consisting of four sections. Section one is to identify if individuals had experienced knee pain and problem in the last year, where if the individuals had experienced knee pain problems, they were allowed to continue the survey. Section two included questions about the clinical characteristics of the knee pain problems to differentiate between PFP and other knee conditions. Section three included questions about painful physical activities associated with knee problems. Lastly, section four included questions to identify the location of the knee pain to discriminate between PFP and other knee pathologies using a knee and patella map.

To obtain the total scores for the survey, Section 2 and Section 4 scores were calculated and combined.

### 2.3. Cross-Cultural Adaptation

Permission was obtained to undertake translation of the SNAPPS from the English language to the Arabic language from the original developers of the questionnaire. The translation process was performed using a forward–backward translation protocol according to the guidelines for the cross-cultural adaptation related to self-report measure translations [10].

At the initial translation stage (stage I), translation into Arabic was conducted by two independent bilingual translators. The first translator had no clinical background, whereas the second translator had a clinical and medical background.

At the synthesis of the translation stage (stage II), the resulting two Arabic translated versions were compared and discussed for any discrepancies. An expert physiotherapist was available to check, ensure clarity, and resolve any discrepancies. The first draft of the Arabic SNAPPS version was produced.

At the back translation stage (stage III), the resulting translated Arabic version was translated back into the English language by two different independent bilingual translators, who both have no medical or clinical background. The resulting back translation was assessed to ensure its similarity to the English version.

At the expert committee stage (stage IV), the expert committee consisted of healthcare professionals, academic methodology experts, and the original authors of the SNAPPS. The committee assessed and ensured all the translations and the original and target version, such as sentences and phrases, are similar.

At the last stage, “pretesting” (stage V), the process included testing the target language questionnaire with a group of 38 participants before finalization of the final version.

### 2.4. Test–Retest Reliability and Validity

To check test–retest reliability, 38 participants filled in the final version of the questionnaire. The questionnaire included information such as age, sex, side of the body with symptoms of PFP, type and frequency of physical activity, and symptoms of PFP. Participants were asked to fill in the questionnaire two times on the same day with a 30 min break in between.

Convergent validity of the questionnaire was assessed by exploring the correlations of the SNAPPS total score with the visual analogue scale (VAS) scores, including VAS for usual pain, VAS for worst pain, and VAS for pain during activities such as jumping, running, ascending and descending stairs, and squatting.

### 2.5. Participants and Sample Size

According to Beaton et al. (2000), a sample size of 30–40 participants is required to achieve r > 0.70, which is considered the lowest border for good retest reliability [10] considering that the correlation coefficient needs to be at least 0.87 (alpha = 0.05; power = 0.05; one-sided) to be accounted for [15].

Participants had no cognitive impairments or neurological disorders. Ethical approval for this study was obtained from the Institutional review board of the Faculty of Allied Medical Sciences at Applied Science Private University (AMS-2024-1-2). All participants provided written informed consent before participating, and all procedures adhered to the principles stated in the Declaration of Helsinki.

The sample size was thirty-eight participants aged 19–30. All study participants were active and performed physical activities (three times per week) without limiting the intensity, type, or duration of each physical exercise session.

Inclusion criteria included participants who are fluent in Arabic, suffering from retropatellar pain for at least three months without traumatic onset, and had increased pain with at least two of the four following activities: squatting, running, jumping, or ascending/descending stairs. Exclusion criteria included participants who were suffering from knee swelling, had characteristics of other knee pathologies, had a history of patella dislocation, or had had knee surgeries within the last six months [2,16].

A physiotherapist assessed and clinically diagnosed participants with PFP with assessment tests including vastus medialis coordination, eccentric step, patellar apprehension, and single leg squat. The diagnosis was confirmed if a minimum of two tests were considered positive.

### 2.6. Patient and Public Involvement

Patients or the public were not involved in the design, conduct, reporting, or dissemination plans of our research.

### 2.7. Statistical Analysis

All statistical analyses were performed using the SPSS statistical package (Version 25.0; SPSS Inc., Chicago, IL, USA). The test–retest reliability of the Arabic version of SNAPPS was assessed using an intraclass correlation coefficient (ICC_3_, 1) with 95% confidence interval. The level of reliability was set as weak reliability (<0.5); moderate reliability (0.5 to 0.7); strong reliability (0.7 to 0.9); and very strong reliability (0.9 to 1.00) [17]. The internal consistency of the Arabic version of the SNAPPS was assessed by determining the Cronbach alpha. The relationships between SNAPPS and VAS U, VAS W, VAS S, VAS J, VAS R, and VAS SQ were assessed using Pearson correlation (*r*) with coefficient values 0.00–0.29 representing negligible correlation, 0.30–0.49 low, 0.50–0.69 moderate, 0.70–0.89 high, and 0.90–1.00 very high correlation [18].

## 3. Results

Overall, 38 participants were included in the study (21 females and 17 males). The average age of the participants was 25.32 (4.14) years. Out of these, 24 participants reported unilateral PFP, and 14 reported bilateral PFP. Means (standard deviations) of the VAS are reported in Table 1.

No major differences between the translators were found for the forward translation step, synthesis step, and the back translation step of the translation process. Only a few words which have similar meanings were different (see Table 2) in step 3 (back translation). A few words were different from the original text because the translators were not experts in medical terminologies, for example, the word “cap” and the word “patella”.

Some words needed to be translated specifically by a healthcare expert or using a medical dictionary (for example, “arthroscopy”, and types of surgeries). The pretest step of the Arabic questionnaire showed that the participants did not have any difficulty in understanding the questions. No questions, comments, or clarification requests were reported by the participants.

The test–retest reliability ICC was 0.92, indicating very strong test–retest reliability of the Arabic version of the SNAPPS (Table 3). The results of the validity test showed that the SNAPPS has a strong correlation with the VAS during ascending and descending stairs (r = 0.71) and moderate correlations during jumping (r = 0.54), and squatting (r = 0.57). The internal consistency of the Arabic version of the SNAPPS was assessed by calculating the Cronbach alpha. The internal consistency was found to be good (Cronbach alpha of 0.84).

Table 4 demonstrates the results of the correlations between the SNAPPS and the visual analogue scale.

## 4. Discussion

SNAPPS was originally designed as a low-cost and easily accessible self-report questionnaire to aid in identifying individuals with PFP within the general population [5]. Since its development in 2016 [5], SNAPPS has been translated and cross-culturally adapted into more than 10 languages [9]. Despite efforts to make it available across different continents, further examinations and cross-cultural adaptations into additional languages, particularly those widely used in the Middle East, such as Arabic, are needed. Consequently, this research was conducted to cross-culturally adapt the SNAPPS questionnaire into Arabic and examine the psychometric properties of the new Arabic version.

Cross-cultural adaptation of any measurement tool is crucial before its use in a new population or language to ensure equivalence between the original and new versions [10]. Therefore, this study followed a well-established cross-cultural adaptation guideline to ensure that the psychometric properties of the Arabic version closely resemble those of the original English version and are suitable for use with individuals affected by PFP. Four translators were involved in the forward and backward translations of the SNAPPS questionnaire. The cross-cultural adaptation method used in this study was similar to that of other studies adapting SNAPPS into the Thai [9] and Chinese languages [8]. The expert committee involved in this study found that the Arabic version of SNAPPS closely resembled the original English version. They identified similarity and equivalence in four different aspects: idiomatic, conceptual, semantic, and experiential. Similar to the Thai version, the Arabic version achieved linguistic equivalence to the English version while considering the Arabic context and maintaining the intent and concept of the original questionnaire. The expert committee found no significant differences between the Arabic and English versions of the questionnaire, except for medical terminology such as translating “Patella” to “kneecap”, a more common term for individuals without a medical background.

Before any assessment tool is utilized in research or clinical settings, its psychometric properties need to be tested [19]. After completing the translation and cross-cultural adaptation process, various statistical tests were conducted to examine different psychometric properties of the adapted questionnaire, such as test–retest reliability, face, and content validity. The test–retest reliability ICC was 0.92, suggesting very strong test–retest reliability of the Arabic version of SNAPPS. The test–retest reliability of the original SNAPPS version ranged from 0.61 to 0.8, considered very good by Landis and Koch [20]. The Thai version of SNAPPS also achieved values greater than 0.9, indicating very strong reliability [9]. Moreover, the internal consistency of the Arabic version of the SNAPPS was found to be 0.8, which is interpreted as a good consistency level according to Bland and Altman (1997) [21].

The validity test findings suggested that SNAPPS has a strong correlation with the VAS during ascending and descending stairs (r = 0.71) and moderate correlations during jumping (r = 0.54) and squatting (r = 0.57). This might be due to knee biomechanics while performing different daily activities. Similar findings were reported by Brady [9]. The forces experienced by the patellofemoral joint, such as the patellofemoral joint reaction force (PJRF), reached 3.3 times the body weight during stair descent. Excessive patellofemoral joint stress (PFJS) is also identified as a contributing factor to patellofemoral pain (PFP). During the controlled descent of stairs, the hip, knee, and ankle joints initially extend and then flex, leading to a progressive increase in external flexion moments. To counteract potential collapse, there is a need for progressively higher levels of eccentric muscle contraction. Previous studies have suggested that knee flexion increases during stair descent, and the patella contact zone shifts proximally, lengthening the patella tendon lever and shortening the quadriceps lever. This shifting contact zone has a notable effect: at knee flexion angles below 60 degrees, the quadriceps lever arm operates with a mechanical advantage, but at angles beyond 60 degrees, the quadriceps functions with a mechanical disadvantage. This creates an intriguing paradox where, as the external moment increases with progressive knee flexion, the demand for increased eccentric quadriceps activity rises simultaneously with decreasing efficiency of the quadriceps. When individuals with PFP descend stairs, the moment arm increases, leading to a heightened pain sensation [22].

During the validation process, several challenges were encountered that are worth noting for future research. One of the main challenges was ensuring that the translated terminology was easily understood by individuals without a medical background. For example, translating specific medical terms such as “Patella” to “kneecap” required careful consideration to maintain accuracy while ensuring comprehensibility. Additionally, cultural differences posed challenges in conveying certain idiomatic expressions and conceptual meanings, necessitating multiple iterations and consultations with bilingual experts to achieve consensus. Despite these challenges, a major strength of the A-SNAPPS was its high reliability and validity, which were comparable to those of the original and other adapted versions. However, one potential weakness observed was the relatively narrow age range of the study participants, which might limit the generalizability of the findings. Furthermore, while the tool showed good internal consistency, the lack of comprehensive testing for content and construct validity indicates the need for further research to confirm these aspects. These insights provide valuable information for researchers and practitioners who aim to apply similar cross-cultural adaptation methods, highlighting the importance of thorough translation, cultural consideration, and extensive psychometric testing.

The research encountered various limitations. The age range of the participants (19–30 years old) did not fully align with the intended scope of the instrument, which targets individuals aged 18 to 40. Further investigation involving diverse populations is necessary to validate and ensure the reliability of the instrument for broader application. Another limitation was that the researcher opted for a 30 min time interval between assessments to accommodate potential fluctuations in pain intensity and for the participants’ convenience. This choice mirrored that in a previous study on the Thai version of the SNAPPS questionnaire, which also used a 30 min interval and demonstrated excellent reliability (ICC = 0.91), consistent with studies from other countries. Given the busy schedules of the study participants, this short interval helped ensure that factors such as physical activity or treatment did not significantly affect clinical conditions. Additionally, it is important to establish correlations between this tool and other instruments related to anterior knee pain. Additionally, the study did not include assessment of content and construct validity of the SNAPPS. Therefore, it is recommended to conduct another study to explore the content and construct validity of the Arabic version of the SNAPPS. Furthermore, comparative validation between the Arabic version of the SNAPPS in Arabic-speaking countries and the existing versions in other languages used in different countries was not conducted due to a lack of data from those countries.

The findings of this study suggest that the Arabic version of the SNAPPS questionnaire is valid and reliable, comparable to the original English version.

## 5. Conclusions

The Arabic version of the Survey Instrument for Natural History, Aetiology, and Prevalence of Patellofemoral Pain was cross-culturally adapted and validated, demonstrating very strong reliability. The Arabic version can now be utilized to assess PFP in adult individuals residing in more than 22 countries across the Middle East and North Africa.

## Figures and Tables

**Table 1:** Results of the visual analogue scale (n = 38).

Symptom of Pain	Mean (SD)
VAS U	3.58 (1.48)
VAS W	6.40 (1.82)
VAS S	4.82 (1.98)
VAS J	5.82 (2.41)
VAS R	5.32 (1.34)
VAS SQ	5.95 (2.97)

Visual analogue scale for usual (VAS U), worst pain (VAS W), and pain during activities (VAS activities) such as ascending and descending stairs (VAS S), squatting (VAS SQ), running (VAS R), and jumping (VAS J).

**Table 2:** Differences in wordings between back translation and original version of the SNAPPS.

Original Text	Back-Translated English Version of the First Translator	Back-Translated English Version of the Second Translator
Have you ever had a kneecap that has gone out of joint (dislocated)?	Have you ever had a patella that has moved out of joint?	Have you experienced kneecap dislocation?

**Table 3:** Mean and standard deviation of test–retest of the SNAPPS, and test–retest reliability results.

SNAPPS	Mean (Standard Deviation)		ICC	95% Confidence Interval	
	First Time	Second Time		Lower Bound	Upper Bound
Total score	8.56 (1.82)	8.48 (1.53)	0.92	0.82	0.96

**Table 4:** Results of the correlations between the SNAPPS and the visual analog scale.

Variables	Correlations					
	VAS U	VAS W	VAS S	VAS J	VAS R	VAS SQ
SNAPPS total score	0.079	0.127	0.71 **	0.54 *	0.273	0.57 *

Visual analogue scale for usual (VAS U), worst pain (VAS W), and pain during activities (VAS activities) such as ascending and descending stairs (VAS S), running (VAS R), jumping (VAS J), and squatting (VAS SQ). * Correlation is significant at the 0.05 level. ** Correlation is significant at the 0.005 level.

## Data Availability

Dataset available on request from the authors. The raw data supporting the conclusions of this article will be made available by the authors on request.
